# The Significance of a Cerebrovascular Accident Outcome Prediction Model for Patients, Family Members, and Health Care Professionals: Qualitative Evaluation Study

**DOI:** 10.2196/56521

**Published:** 2025-01-22

**Authors:** Corinne G Allaart, Sanne van Houwelingen, Pieter HE Hilkens, Aart van Halteren, Douwe H Biesma, Lea Dijksman, Paul B van der Nat

**Affiliations:** 1 Department of Computer Science Faculty of Science Vrije Universiteit Amsterdam Amsterdam Netherlands; 2 Department of Value Improvement St. Antonius Hospital Nieuwegein Netherlands; 3 Philips Research Eindhoven Netherlands; 4 Leiden Unversity Medical Center Leiden Netherlands; 5 IQ Healthcare Radboud University Medical Center Nijmegen Netherlands; 6 Santeon Utrecht Netherlands

**Keywords:** cerebrovascular accident, machine learning, artificial intelligence, visualization, focus groups, questionnaire, informal caregivers, health care professionals

## Abstract

**Background:**

Patients with cerebrovascular accident (CVA) should be involved in setting their rehabilitation goals. A personalized prediction of CVA outcomes would allow care professionals to better inform patients and informal caregivers. Several accurate prediction models have been created, but acceptance and proper implementation of the models are prerequisites for model adoption.

**Objective:**

This study aimed to assess the added value of a prediction model for long-term outcomes of rehabilitation after CVA and evaluate how it can best be displayed, implemented, and integrated into the care process.

**Methods:**

We designed a mock-up version, including visualizations, based on our recently developed prediction model. We conducted focus groups with CVA patients and informal caregivers, and separate focus groups with health care professionals (HCPs). Their opinions on the current information management and the model were analyzed using a thematic analysis approach. Lastly, a Measurement Instrument for Determinants of Innovations (MIDI) questionnaire was used to collect insights into the prediction model and visualizations with HCPs.

**Results:**

The analysis of 6 focus groups, with 9 patients, 4 informal caregivers, and 8 HCPs, resulted in 10 themes in 3 categories: evaluation of the current care process (information absorption, expectations of rehabilitation, prediction of outcomes, and decision aid), content of the prediction model (reliability, relevance, and influence on the care process), and accessibility of the model (ease of understanding, model type preference, and moment of use). We extracted recommendations for the prediction model and visualizations. The results of the questionnaire survey (9 responses, 56% response rate) underscored the themes of the focus groups.

**Conclusions:**

There is a need for the use of a prediction model to assess CVA outcomes, as indicated by the general approval of participants in both the focus groups and the questionnaire survey. We recommend that the prediction model be geared toward HCPs, as they can provide the context necessary for patients and informal caregivers. Good reliability and relevance of the prediction model will be essential for its wide adoption.

## Introduction

Cerebrovascular accident (CVA), the collective name for ischemic stroke and intracerebral hemorrhagic stroke, affects 15 million people each year globally [1]. A CVA can have a range of symptoms that vary from patient to patient. Unilateral paralysis or weakness, numbness or loss of vision, speech difficulties and other cognitive dysfunctions, ataxia, diplopia, and nonorthostatic dizziness are some of these symptoms [2]. After the acute phase of treatment, patients often undergo a long rehabilitation process, although the length, intensity, and outcome can vary greatly between patients. In the Netherlands, 65% of CVA patients undergo rehabilitation at home after their hospitalization. The remaining 35% of CVA patients visit a rehabilitation center or geriatric rehabilitation center. Among patients in this group, 25% will not return to their homes and will go to nursing homes [3].

To provide the best care, health care professionals (HCPs) and patients should decide together which path to take in the care process [4]: shared decision-making. Efficient and practical goals can be determined when patients are involved in decision-making, leading to a more appropriate and suitable discharge location. Thus, CVA patients should be involved in decision-making regarding where rehabilitation will take place and setting rehabilitation goals [5]. A considerable number of CVA patients prefer active involvement in decision-making and consider themselves capable of doing so [6]. An important part of supporting active involvement in the decision-making process is to offer patients insights into expected outcomes.

Recovery is very heterogeneous for CVA patients in terms of speed, process, and outcomes, and is often considered too complex for HCPs to predict. As such, little information about expected medium- and long-term outcomes (3 months to >1 year) is provided to patients. Over the last few years, using artificial intelligence (AI) methods, several outcome prediction models have been developed. Some models on certain aspects of functional status have been developed, such as rehabilitation of motoric upper limb skills [7] or independent walking [8]. Others have reported testing at rehabilitation admission [9] or the use of wearable sensors [10] to provide useful data to predict postrehabilitation CVA outcomes. Most of these models use machine learning algorithms on structured clinical data, with varying accuracy [11,12]. Deep learning has offered the opportunity to combine more data and more different data types, such as imaging or free text, into a prediction model [13] and as such increase the accuracy of predictions. Several studies have investigated how deep learning can lead to better CVA outcome predictions [13,14] to reach clinically acceptable reliability. One of our previous studies [15] showed that combining structured clinical data and perfusion computed tomography (CT) scans can lead to an increase in performance.

While having accurate and reliable predictions is important, the explainability and clinical relevance of AI methods are essential for their adoption in medical practice [16,17], and many prediction model implementations currently fall short in these aspects [18]. Using a functional tool based on AI in a real-world setting would require both HCPs and patients to understand and trust the outcome prediction and see its relevance. Understanding a predicted outcome can be challenging for healthy people, but even more for CVA patients. These patients can have impaired cognition and often have a higher age [2]. Thus, information should be provided in an easy and clear manner for low cognitive effort. For example, when numerical data are presented, it is recommended to use simple visual forms such as flow or bar charts and circle diagrams [19].

The discharge interview usually addresses the duration and location of the rehabilitation. There are typically multiple possibilities after discharge, such as going home without or with home therapy, outpatient rehabilitation, geriatric rehabilitation, and clinical rehabilitation treatment [3]. A personalized prediction of CVA outcomes and the length of the rehabilitation process would allow HCPs to better inform patients and informal caregivers. This could form a basis for shared decision-making, as a more informed conversation about the preferences of the patient can be held. This research aims to assess the added value of a prediction model for long-term outcomes of rehabilitation after CVA, to investigate how its results can be best displayed, and to determine the best way to implement and integrate it into the care process.

## Methods

### Design

A qualitative study was designed with the goal of evaluating the added value of a prediction model for stroke outcomes in CVA care. More specifically, this study aims to investigate how to best display the outcomes of the model, and integrate and implement them in the care process in an appropriate manner. We based our study on a prediction model previously designed in our hospital [15]. Before the qualitative evaluation, the research team developed a mock-up of a display of the prediction model, together with several different visualizations of the model outcome and interpretability. The research team included a neurologist and an expert on medical AI. The mock-up design was iterative, allowing for an update after each part of the study.

**Figure 1:**
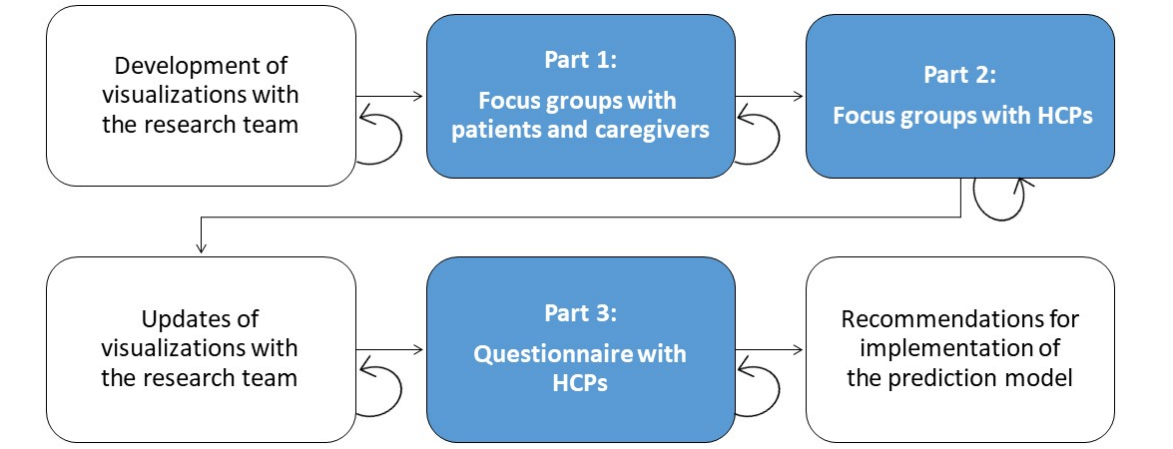
Schematic overview of the different parts of the study. The circular arrow refers to the iterative aspect of each step. HCP: health care professional.

The study consisted of 3 parts: focus group discussions with CVA patients and their informal caregivers, focus group discussions with HCPs, and a follow-up questionnaire to HCPs, as shown in Figure 1. In both sets of focus groups, the opinions of patients and HCPs on the current information management and their needs with regard to the prediction model were explored and analyzed using a thematic analysis approach. The questionnaire was used to evaluate the finalized prediction model and visualizations with the HCPs.

### Focus Groups

#### Study Population

Patients who were admitted to St. Antonius Hospital between January 2020 and December 2021 for a CVA were selected using convenience sampling. Patients who had a CVA in the previous 3 months or who did not speak Dutch were excluded. We selected participants with a wide variation in age, gender, rehabilitation type (medical specialist, geriatric, home, or outpatient), and education level. An overview of the characteristics of the selected participants can be found in Table 1. Patients were approached by phone, and information letters were sent by email or postal address. Patients could bring their informal caregivers to the focus groups if this was of added value due to impaired cognition or higher age. The research team thought that an informal caregiver could assist in interpreting the model and could discuss patient preferences. Another advantage of including informal caregivers is that they are often involved in the entire hospitalization process of patients, and their opinions can therefore be valuable. Participants signed up by contacting the researcher and were asked to sign an informed consent form before the focus group started.

**Table 1:** Cerebrovascular accident participants in focus groups.

Participant	Age range (years)	Gender	Level of education	Rehabilitation
P1	71-75	Female	Vocational education	Geriatric rehabilitation center
P2	41-45	Male	Vocational education	Rehabilitation at home
P3	41-45	Female	Vocational education	Rehabilitation center
P4	71-75	Male	Applied university	Rehabilitation at home
P5	81-85	Female	High school	Rehabilitation at home
P6	66-70	Female	Applied university	Rehabilitation at home
P7	41-45	Male	High school	Rehabilitation at home
P8	76-80	Female	Vocational education	Geriatric rehabilitation center
P9	61-65	Female	Vocational education	Rehabilitation center

For HCPs, participants were recruited from a pool of HCPs involved in the discharge or rehabilitation process in the hospital. This included neurologists, rehabilitation doctors, junior doctors, nurses, and nurse specialists. All participants were approached via email or in person. The research team aimed to have 3 participants in each focus group, specifically a medical specialist, a general doctor, and a nurse.

#### Data Collection

The focus groups were led by 2 researchers (CGA and SvH). SvH moderated the focus groups with patients, and CGA moderated the focus groups with HCPs. We opted for the separation of patients and HCPs in different focus groups, such that the participants in both groups would be able to speak more freely about their experiences. We started with 3 focus groups for patients and their informal caregivers and 3 focus groups for HCPs, and we assessed whether saturation of data was reached or more focus groups were necessary. Each focus group had different participants.

The focus groups consisted of 2 phases. The first phase of the focus group was intended to explore the needs and expectations of the use of a prediction model during the discharge interview. This started with a reflection on the discharge interview and rehabilitation process, what expectations were created, what shared decisions were made, how the process went, and other questions about topics and components that were addressed in the prediction model. The goal was also to determine how a prediction model could contribute to better expectations and improve the discharge interview.

In the second phase of the focus groups, different options for visualizations of the prediction model were discussed. We have elaborated on their design in the section on visualizations. Before the second phase of the focus group started, there was a short explanation of the visualizations. After this, the participants were asked to discuss several topics. First, whether the visualizations were clearly explained. Second, they were asked about relevance and what they think about the visualizations being used. During the focus groups with HCPs, this also included the predictors included in the model. Finally, they were asked to discuss which of the visualizations they preferred and why.

#### Focus Group Guide

Focus group guides were used, consisting of open and follow-up questions for the 2 phases of the focus groups (Multimedia Appendix 1 for patients and Multimedia Appendix 2 for HCPs). The focus group guides consisted of a few open questions, aimed at starting a discussion on information provision to patients in the current care process and the suitability of the prediction model and its visualizations. The focus groups followed an iterative design, which meant that after each focus group, the questions in the interview guide were adjusted if certain topics needed to be added or changed.

#### Data Analysis

Analysis of the focus groups was performed by researchers CGA and SvH in a thematic analysis approach using Atlas.ti [20]. CGA and SvH are early stage researchers with educational training and some research experience in focus group research. The thematic analysis was performed as follows. First, the researchers transcribed and anonymized the interviews. Then, both researchers separately open coded the transcripts. The data were coded axially by clustering codes by meaning, and the clusters were assigned themes. When all 6 transcripts were axially coded, the data were selectively coded to determine the relevance and consistency of the themes [21]. Finally, the themes were defined by noting the characteristics of the themes for consistent use. After analysis, the researchers held discussions about the data until a consensus was reached. We aimed for research triangulation by having 2 researchers independently analyze the data and iterate until agreement.

### Questionnaire Survey

#### Study Population

The study population of the questionnaire consisted of HCPs. Similar to the focus groups, the participants were recruited from the pool of HCPs involved in the discharge or rehabilitation process in the hospital. However, only HCPs who directly worked with the prediction mode, such as junior doctors and neurologists, were asked to fill out the questionnaire. They were contacted by email or phone, or in person.

#### Data Collection

The last part of this study consisted of a questionnaire for a final analysis of the visualizations by HCPs. A simulated visualization (updated after the focus groups; see section visualizations) was shown to the HCPs for different scenarios for possible situations of patients in a discharge interview. They were asked to answer a modified Measurement Instrument for Determinants of Innovations (MIDI) questionnaire [22], which is a tool for describing the helpfulness and possibilities of medical technological devices. We selected 2 relevant sections out of 4 in the questionnaire regarding the innovation itself and the end users. The other 2 sections regarding work environment and sociopolitical environment were out of scope and more suitable for a later implementation phase. The questionnaire was adapted to fit the setting of the prediction model and was distributed via RedCap [23]. The questions were divided into 5 sections: general opinions, advantages and disadvantages, effects on patients, effects on colleagues, and opinions on implementation. The questionnaire consisted of 21 questions on a Likert scale and 1 open question.

#### Data Analysis

No statistical analyses were performed on the questionnaire, as the MIDI questionnaire is not a validated questionnaire and the sample size is small. Instead, the questions were meant to provide a formalized evaluation of the prediction model and its visualizations. Both the general score of the prediction model and the agreement have been reported.

### Prediction Model and Visualizations

The model visualizations were based on the model described in a previous article [24]*.* This model aims to predict a 2-fold outcome: functional outcome (modified Rankin scale [mRS] score) after 3 months and length of stay in a rehabilitation center. Two feature sets were used as predictors: hospital data and data from geriatric rehabilitation clinics. The hospital data were based on registry data from the Dutch Acute Stroke Audit [25], such as medical history, treatment data, and admission data, and the other data included intake data, admission data, and discharge data.

**Figure 2:**
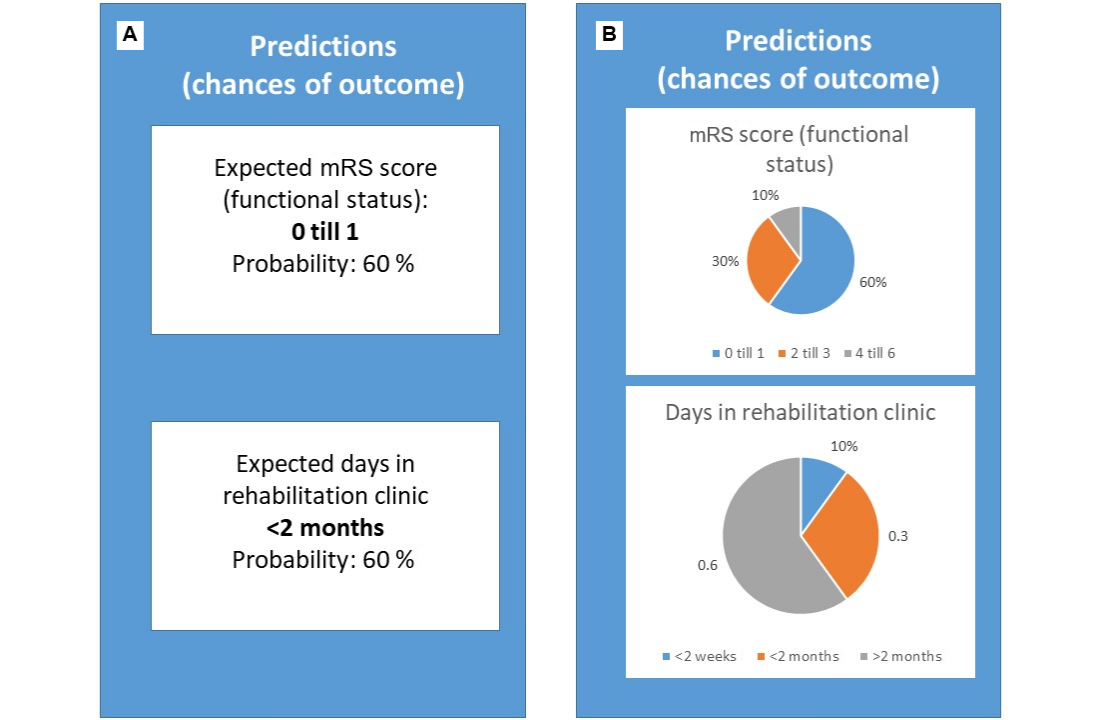
Different visualizations of outcomes (translated from Dutch). (A) Textual limited information; (B) Visual detailed information. mRS: modified Rankin scale.

The design of the visualizations followed an iterative setup with the research team. Before the focus groups, the research team created a mock-up, with outcomes and visualizations for 2 fictional patients, as a functional model with real patient data was not yet available. Based on examples and previous literature about explainable AI in health care [26,27], a choice of 2 different visualization styles was made. One with a more simplified textual approach having clear and limited information, and one with a more detailed visualized approach having more information in the form of diagrams. We chose circle and bar charts for this style, as they are considered the most “approachable style” of visualization of numbers [19]. The visualizations of the outcomes in both styles are shown in Figure 2.

**Figure 3:**
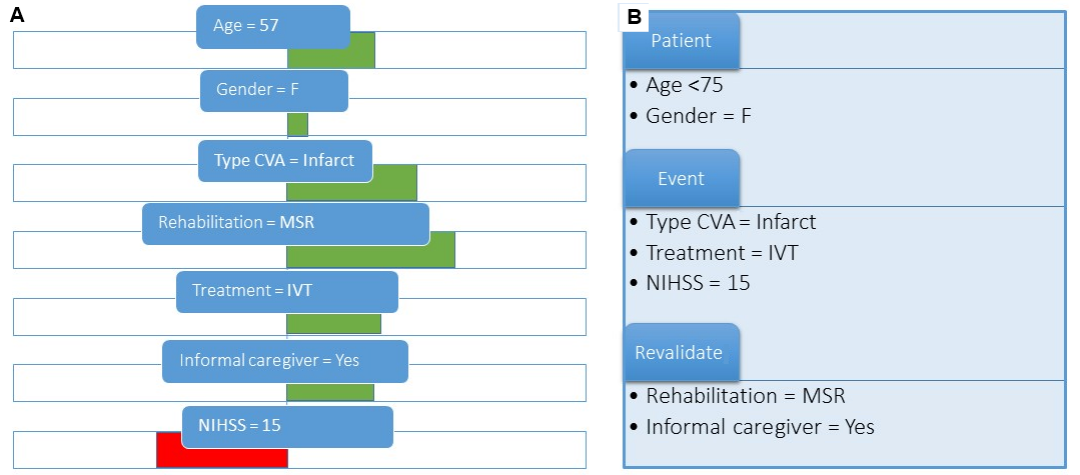
Different visualizations of influencing factors (translated from Dutch). (A) Visual detailed information. The color and length of the bars indicate the size of the effect on the outcome. (B) Textual limited information. CVA: cerebrovascular accident; IVT: intravenous thrombolysis; MSR: medical specialistic rehabilitation; NIHSS: National Institutes of Health Stroke Scale.

Next to outcome visualizations, we also included explanations of the predicted outcomes by the models, which have been shown to be important for the adoption of AI in medicine [14]. We opted to show the model explainability through feature importance, where for the predicted outcome of the model for each patient, the contribution of each feature is shown. This can also be described as local interpretability, which has been further explained previously [19,23]. For example, it can show that the age of that specific patient contributed 20% to the decision of their personal outcome. This can be both positive or negative feature importance, indicating whether it made the predicted outcome more or less likely. The detailed visualization (Figure 3A) shows this in red and green bars. For the simplified version (Figure 3B), only the most important features for the outcome were mentioned (not the extent of their contribution).

Visualizations were developed for 2 fictional patients in 2 different visualization styles, resulting in 4 examples. The complete translated visualizations can be found in Multimedia Appendix 3. Based on the feedback from the focus groups, 1 visualization style was selected, and the visualizations were modified in accordance with the focus group feedback. These final visualizations were used for the questionnaire.

### Ethical Considerations

The Medical Ethics committee of Utrecht (MEC-U) declared that this study is not subject to the Dutch Medical Research Involving Human Subjects Act (WMO) and issued a non-WMO statement under number W22.007, and subsequently, the board of directors of St. Antonius Hospital issued a statement of no objection (permission to perform) under number Z22.009. All participants provided informed consent.

## Results

### Focus Groups

The focus groups took place in April and May 2022 and lasted for 60-90 minutes with patients and caregivers and for about an hour with HCPs. We conducted 3 focus groups of each type (total 9 patients, 4 informal caregivers, and 8 HCPs). After these sessions, further focus groups were not deemed necessary, as saturation was reached.

A few minor changes were made to the interview guide after the first focus group with patients. We put less focus on the discharge interview and more on the questions about the care process in general for patients and the family interview for HCPs. For the focus groups with patients and informal caregivers, the transcripts were sent to these participants for a member check. None of the participants had any remarks on the transcripts.

**Figure 4:**
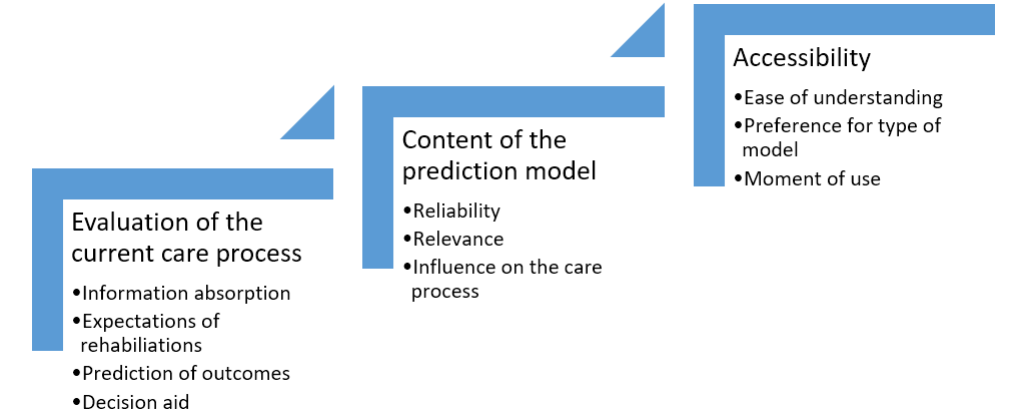
The 10 themes in the focus groups divided into 3 categories.

The results of the focus groups have been organized by theme in prediction model development in 3 sections: evaluation of the current care process, content of the prediction model, and accessibility of the model (Figure 4).

#### Evaluation of the Current Care Process

##### Information Absorption

Some of the patients said that they either could not remember or did not understand the information given at the discharge interview. This could be due to the symptoms of their CVA. For example, a patient developed aphasia because of the CVA. As a result, she did not know what questions to ask during the discharge interview and did not manage to do so. However, most patients said this was due to information overload caused by discharge taking place shortly after the CVA, and the impressions and emotions during admission. On the other hand, most of the informal caregivers said that they received the information provided during the discharge interview and helped the patients with the information when necessary.

The patients and informal caregivers who could remember said that they were positive about the information provision, calling it clear, open, and informative, with a lot of room for questions. HCPs mentioned that they often found it hard to estimate whether patients and caregivers truly understood the given information, saying that patients always say they understand, even if they do not. They also mentioned language barriers as a common issue. Moreover, they were not sure whether the extra resources given, such as websites or information flyers, were read and understood by patients, as they did not discuss these with them.

##### Expectations of Rehabilitation 

Most patients and informal caregivers had little idea of what to expect from rehabilitation and expressed that they felt thrown in at the deep end. One patient described her lack of expectations as follows:

I just let everything wash over me. That was all I could do.

Only 1 patient said he knew what he could expect in his personal situation, even if daily tasks proved more difficult. Informal caregivers expressed wishes for more information from the hospital about rehabilitation, with 1 informal caregiver expressing that he would have wanted a rehabilitation plan from the hospital:

[...] my mom is not the first one with a CVA, so there is a certain expectation and based on that […] protocols are made. It's my first time, and then there's nothing […] that's hard. Something has to be done about that. That's what I missed.

Eventually, most patients were happy with the rehabilitation, except for a few bad experiences with HCPs. HCPs’ experiences matched with the views of the patients and caregivers, and they stated that they do not have a good overview of rehabilitation and therefore cannot give patients an expectation of what will happen during rehabilitation. Some HCPs believed that this information should be given in rehabilitation clinics. They also mentioned that there could be a disparity in the expectations between patients and informal caregivers. In those situations, patients often felt like they were ready to be discharged home, while caregivers would still be very worried and push for inpatient rehabilitation.

##### Predictions of Outcomes

Almost all HCPs mentioned the difficulty of making individual predictions. They explained that this was due to patients, even similar patients, having varying recovery paths and because they did not have oversight over recovery during rehabilitation. Only the 2 most experienced neurologists felt more comfortable giving general predictions using age and the severity of the event. One of the junior doctors mentioned that they did not feel like they had enough knowledge and data to make such predictions.

I also don't know the exact scientific evidence from the top of my head […] for someone with a hemiparesis after 3 months.

These statements were in agreement with the experiences of the patients. In the perception of the patients, if the physician did predict outcomes, it was not very precise. For example, they mentioned that the physician predicted that they would recover gradually in the first 3 months. Moreover, they said that no data in the form of numbers or percentages were used. 

We asked all participants what they considered the most important outcomes to have a prediction on. Three outcomes were mainly mentioned: functional status in the medium or long term (multiple months or years), time of rehabilitation, and the chance of a new CVA. A new event was something that many patients were very scared about. They wanted to know how high the chance of recurrence was and wanted reassurance that it was as low as possible. HCPs mentioned it was not necessary to have a prediction of recurrence, as medications will always be given to minimize those chances. 

##### Decision Aid

Many patients and informal caregivers felt like the physician took their opinions into account. According to the participants, it varied for each patient whether they had a choice in discharge location. Most participants said they did not have a choice in this decision. However, most participants felt that they could not make a better decision than a physician could, and they accepted the physician’s opinion and professionalism. According to the participants, physicians have more knowledge about a CVA than patients and therefore know what is better for them.

You have to imagine; it was all new to us. So […] how can your opinion be different from what they recommend to you? It's almost impossible because you're in a situation like that so you can't judge it.

Physicians mentioned shared decision-making. There is often not really any discussion on where the patient should be discharged.

#### Content of the Prediction Model

##### Reliability

Many patients were initially skeptical about a model that would predict outcomes. This was based on the physician’s statement that it is not possible to make personal predictions. Several patients and informal caregivers said they did not believe that a physician could make accurate predictions and that consequences could only be seen after rehabilitation. Furthermore, some caregivers also found the prediction too absolute and programmed.

If it’s such a well-founded prediction but we asked the neurologist about the whole thing, and he couldn't really give us any answers about predictions […]. Won’t we get the wrong expectations from an overview like this?

HCPs were also skeptical of the reliability of the model. They similarly argued that they see a lot of variation between patients and wondered if it is possible to make accurate predictions. In general, they believed that all included predictors would be usable. However, the HCPs questioned the reliability of the model due to the absence of certain important predictors, such as pre-existent functioning (Barthel score), detailed comorbidity, and the character of the patient. They acknowledged that the last aspect would be hard to measure. Moreover, if it is just a tool for information, the doctors argued that it is not that bad if the prediction is not 100% accurate**.**

[perfect performance] would be preferable, but you can never predict that […] maybe the important part is how to translate model performance to the patient

One of the informal caregivers felt that there should be a disclaimer with the model because the outcome could never be 100% right.

##### Relevance

While most HCPs were generally enthusiastic about the proposed model, another point of skepticism was regarding relevance, as they were hesitant about how to explain the predicted outcomes to patients, especially regarding percentages. Patients confirmed these concerns. One of the patients said he did not like percentages because one can never say what 90%, for example, truly quantifies. Patients agreed that it is better to have a conversation and explain the information instead of simply showing the model. HCPs mentioned that while a general model for outcome prediction would be good, a more useful option would be if there were more different outcomes. For example, where it would be more useful if we could split it into several types of complaints or symptoms (like aphasia or paralysis).

There was disagreement among patients regarding whether they wanted predictions at all. Some stated that they would be curious to know their predictions. Another participant stated that the information he received from his physician was sufficient and that he did not need a more specific prediction. Some of the caregivers said they would find it convenient to use a prediction model during a discharge conversation and that a prediction could supplement the information given by HCPs. Most HCPs were slightly worried about the risk of giving too much information, as it is currently already too much. However, some physicians also thought that a prediction could help clarify the situation, making the other information more digestible.

##### Influence on the Care Process

There were several points discussed concerning the possible influence of a prediction model on the care process. There was agreement among HCPs that it would be good for the patient as guidance if it is understandable for the patient. Junior doctors especially thought it would be good for HCPs as well for guidance in conversations with patients, reaching a better ground to make prognoses and overcoming unconscious biases about patients.

While most patients and informal caregivers expressed the positive aspects of guidance similar to HCPs, some patients worried that if the prediction is negative, it could be scary or it could demotivate the rehabilitation process. The patients stated that a lot of motivation is necessary for the best recovery.

And if I were [example patient] and 82 [years old] I don’t know if I would be up for knowing […] that bad outcome

However, most patients and informal caregivers did not want HCPs to hold back negative results from them. Moreover, doctors mentioned they would not be worried about this, as they are used to delivering bad news.

The main importance for HCPs was a way to inform patients and to offer clearer information for expectation management in the further rehabilitation process. As such, using this prediction tool would not have a direct result on the treatment or outcome of patients. There was the question of why this would be useful, and it was expressed that the goal of the tool should be clear; otherwise, it might not be used.

#### Accessibility

##### Ease of Understanding

The HCPs in general found the prediction model and the visualizations quite clear, and most thought that with explanation the model should be understandable for patients. However, several patients and informal caregivers found it hard to understand. Even after another explanation, they struggled to fully comprehend the meaning. One patient mentioned this might be due to one of the complications of his CVA being a lack of concentration and thus not having enough concentration to read. They struggled with the medical terminology, even after explanation. One patient said that it might be convenient for a physician but it is too hard for a patient. Most patients and caregivers understood the visualizations if they looked at them for a little longer but concurred that an explanation is needed from someone with a medical background or a more detailed but simple explanation is needed in additional text.

##### Preference for a Model Style

There were differences among patients regarding the preferences for the style of visualizations. Most of the participants preferred the prediction model with more graphics and less text because it was more visual and easier to understand. One participant stated that when a patient has symptoms like trouble with reading or concentration, it is easier to see the graphs.

I also think that for people who have […] more symptoms, who understand it a […] less easily […] the pictures are a little bit more informative.

A few patients said the bar charts for feature importance did not have their preferences, as they contained too much information and the features could not be changed.

All HCPs preferred the more detailed visualization, and they argued that they contained more information and made the prediction more “alive.” Interestingly, they thought patients would not have the same preferences. The HCPs also argued for integration into the electronic health record (EHR), as this would help with ease of use and adoption in the care process. Some HCPs were afraid that due to the high workload and the model not having a direct impact on the care process, the model might not be used if it is not easily accessible.

##### Moment of Use

Whether to use the model during the discharge interview or during the family interview, which happens a few days into admission with informal caregivers, was a point of discussion among HCPs. It could be beneficial for both. The discharge interview can be very rushed, and during the family interview, there might be more time to go over the predictions. However, this would be earlier in the process, which means that less information might be included in the predictions. Moreover, they agreed that having some form of access afterward would be good. Different options were mentioned: at the rehabilitation center, with the general practitioner, or at the 3-month follow-up.

The patients generally agreed with this view. One patient stated that it would be good to see the prediction when you feel like it, but it would have been too much during the discharge interview.

It is useful but […] too extensive [for] a normal person […] does not understand a thing. [...] Then it is explained by someone with a medical background […] who can perhaps explain it better. […] Maybe after a few months go back and look at it again […]. What are the expectations now, what are the expectations later.

### Questionnaire Survey

Based on the results of the focus groups, we extracted a set of recommendations. These recommendations were either immediately included in the visualizations of the prediction model or served as general recommendations for further development or for the context of the implementation in current care. The recommendations are summarized in Table 2.

**Table 2:** Recommendations from the focus groups.

Category	Theme	Recommendation	Incorporation
Evaluation of current care	Information absorption	Recognize that the information might not be understood at the discharge interview	Implementation
Evaluation of current care	Expectations of rehabilitation	Create a better overview of rehabilitation for patients, informal caregivers, and health care professionals	Context of care
Evaluation of current care	Prediction of outcomes	Highlight that the model meets the needs of patients, informal caregivers, and health care professionals regarding providing a prediction of the outcome	Immediately
Evaluation of current care	Decision aid	Make sure the patient's opinion is heard in the decision process, for example, through shared decision-making	Context of care
Accessibility	Ease of understanding	The target audience of visualizations should be health care professionals and not patients	Immediately
Accessibility	Moment of use	Do not limit the use of prediction to the discharge interview	Implementation
Accessibility	Model preference	Visualize outcome and feature importance (visualization version b)	Immediately
Content of the model	Reliability	Include the general predictive performance of the model, for example, according to the margin of error	Future research
Content of the model	Relevance	mRS^a^ score in 3 categories, including the possible functional score, measured during hospital stay	Future research
Content of the model	Influence on the care process	Clarify the goal of the model (informing patients) to improve adherence	Immediately

^a^mRS: modified Rankin scale.

The visualizations were adapted according to the recommendations. We selected the more “visualized” versions (see Figures 2B and 3A). Moreover, we focused on HCPs as our target audience and therefore removed explanations of well-known abbreviations and concepts, such as the National Institutes of Health Stroke Scale (NIHSS) score. We also specified that the moment of use could be the discharge or family interview and that the goal of the prediction model was to inform patients. Moreover, we included more detailed information on feature importance, based on our underlying model. The adapted visualizations can be found in Multimedia Appendix 3.

The questionnaire was sent to 16 relevant HCPs in St. Antonius Hospital, and we received 9 responses. The answers to the closed questions are summarized in Table 3. For each question, we have reported scores. Moreover, we have presented the average score and SD for each section. Negatively phrased questions (questions 4, 8, and 9) were scored in reverse.

**Table 3:** Questionnaire results reported on a Likert scale (0-5).

Question^a^			Score, mean (SD)
**General statements**			
	1. The prediction model matches well with my usual way of working.	4.0 (0.5)	
	2. The prediction model is based on factually correct information.	3.7 (0.5)	
	3. The prediction model offers all the information and materials necessary to work with it properly.	3.6 (0.5)	
	4. The prediction model is too complicated for me to use.	4.1 (0.8)	
	5. I expect that the effects of the use of the prediction model will be clearly visible.	2.9 (0.4)	
	6. I think the prediction model is suitable for my patients.	3.8 (1.0)	
**Advantages and disadvantages**			
	7. The use of the prediction model gives me the opportunity to inform my patients better.	3.8 (1.0)	
	8. The use of the prediction model during discharge or family interviews will cost me more time than usual.	3.2 (0.5)	
	9. I find it too complicated to use the prediction model during discharge or family interviews.	3.9 (0.8)	
**Prediction model and patients**			
	11. I expect that with the prediction model, my patients will be better informed about the expected functional status after 3 months and the duration of rehabilitation.	3.4 (0.5)	
	12. I think it is important to use the prediction model to better inform my patients about the expected outcomes: the functional status after 3 months, and the duration of rehabilitation.	4.0 (0.5)	
	13. I consider it part of my job to use the prediction model.	3.6 (0.7)	
	14. Patients will generally be satisfied if I use this prediction model.	3.8 (0.4)	
	15. Patients will generally be cooperative if I use this prediction model.	3.7 (0.5)	
**Prediction model and colleagues**			
	16. I can count on sufficient help from my colleagues when necessary while using the prediction model.	3.5 (0.5)	
	17. How many doctors who conduct discharge interviews will actually use this prediction model?	3.8 (0.7)	
	18. To what extent will (your fellow) neurologists expect you to use the prediction model?	3.2 (0.4)	
	19. When it comes to working with the prediction model, how much do you care about the opinion of (your fellow) neurologists?	3.7 (0.5)	
**Application of the prediction model**			
	20. If you wanted to, do you think you would be able to apply the prediction model during the discharge or family discussions?	3.9 (0.3)	
	21. I have sufficient knowledge to be able to use the prediction model.	3.8 (0.8)	
	22. To what extent are you aware of the content of the prediction model?	3.1 (1.1)	

^a^Question 10 was an open question (What are the other advantages or disadvantages of this prediction model?) and is not included in the table.

We considered statements with a score of ≥3.5 and an SD of <1.0 to be sufficient to positive, with agreement among HCPs. Most statements were received as such, and we considered the general feedback to be moderately positive. Moreover, in all categories of statements, we noted that most statements were at least moderately positive with agreement, highlighting that there is no topic where the prediction model consistently underperforms. The high scores for “*The prediction model matches well with my usual way of working*” (score 4.0), “*I think it is important to use the prediction model to better inform my patients about the expected outcomes: the functional status after 3 months, and the duration of rehabilitation*” (score 4.0), and “*If you wanted to, do you think you would be able to apply the prediction model during the discharge or family discussions?*” (score 3.9) indicate a high willingness to implement the prediction model.

For some questions/statements, the HCPs were not positive on average (score of <3.5) or were not in agreement (SD ≥1.0). The statement “*I expect that with the prediction model, my patients will be better informed about the expected functional status after 3 months and the duration of rehabilitation*” had a score of 3.4. This was just under our set threshold. Interestingly, the statement “*I think it is important to use the prediction model to better inform my patients about the expected outcomes: the functional status after 3 months, and the duration of rehabilitation*” had a score of 4.0. It shows that the HCPs, even when not sure about the positive outcome of use, still found it important to use the prediction model. The same concern was highlighted in 2 statements that had high disagreement among the HCPs: “*I think the prediction model is suitable for my patients*” (score 3.8, SD 1.0) and “*The use of the prediction model gives me the opportunity to inform my patients better*” (score 3.8, SD 1.0). This shows consistency with the focus groups indicating that there seems to be variety in the extent to which HCPs are positive about the effects of the prediction model.

In the other statements that were not positive on average or had high disagreement, we also noted similar findings that came up in the focus groups as well. The statement “*I* *expect that the effects of the use of the prediction model will be clearly visible*” had the lowest score of 2.9. This is not completely unexpected as the model’s primary function is to inform and not provide decision support. Moreover, the issue of high workload was reflected in the statement “*The use of the prediction model during discharge or family interviews will cost me more time than usual*” (score 3.2). Lastly, the statement “*To what extent are you aware of the content of the prediction model?*” had both a low score and high disagreement (score 3.1, SD 1.1), indicating that more detailed explanation and training are necessary to understand the model.

The statement “*To what extent will (your fellow) neurologists expect you to use the prediction model?*” had a low score of 3.2. This shows that many HCPs would consider the use of such a prediction model to be more elective. Next to the scaled questions, we also asked 1 open question “*what are the other advantages or disadvantages of this prediction model?*” A big point of contention was whether the prediction model would be accurate enough, as certain factors like recovery during the time in the hospital and mental resilience were not included. A positive aspect mentioned a few times was that it could also be used to preinform the rehabilitation clinics for helping with planning.

## Discussion

### Principal Findings

This study shows the added value of a prediction model for long-term outcomes of rehabilitation after stroke in CVA care under certain conditions. The need for a prediction model was consistently shown in focus groups and a questionnaire survey, as evidenced by the general approval of participants. Several recommendations can be made, most importantly on the following 5 conditions: primary target, timing of discussing the predictions, different focuses of HCPs and patients, goal of the prediction model, and visualization of the model.

First, the primary target audience of the prediction model should be HCPs. While the information provided is of interest to both patients and informal caregivers, not all patients can fully understand the outcomes of the prediction model. This resembles the findings of other studies that investigated different digital health tools for stroke patients, which reported complaints of difficulty [28] or disinterest among some patients [29]. Our study specifically found that patients prefer HCPs to provide information and guide them through the visualizations of the model.

Second, the timing for using predictions should also be considered. As patients mentioned, it could be more useful when targeted toward patients at different points in the care process. Here, it is interesting to see if the model can be simplified, as was requested by interested patients, and how that should be offered. Another study on digital health tools for patients with brain injuries [30] highlighted the importance of user testing among these patients. This would be a good way to ensure that the simplified model fits the patient’s needs and understanding.

Third, for HCPs, the reliability and relevance of the model are key. HCPs focus largely on the reliability and relevance of the model. It is essential to not only provide good reliability and relevance but also clearly communicate the aspects to HCPs. Reliability is essential to validate the performance of the model. Therefore, changes in outcome reporting were considered to be more in line with the current care [31]: divide the mRS score into 3 categories and include predictive factors. By including the general predictive performance of the model (eg, according to the margin of error), the reliability can be better communicated to HCPs. These 2 points of reliability and relevance are very much in agreement with the guidelines for the implementation of AI in health care [32,33]. While the performance and generalizability of the model were not the focus of this study, they should be properly validated before model implementation. We found that patients focused more on the impact on rehabilitation, the emotional aspects, and understandability.

Fourth, HCPs and patients should understand the goal of the prediction model. This understanding can improve adherence and the willingness of HCPs to cooperate, which are essential for the adoption of the model in practice [32]. This matched our results, as this was one of the main points of the HCPs in both focus groups and the questionnaire survey. We aimed to clarify the goal of the prediction model after the focus groups. However, the questionnaires showed that the goal of the prediction model was still unclear to some HCPs, even after a more detailed explanation in the introduction of the questionnaire. This could be mitigated by properly informing HCPs, for example, by providing in-person training or a detailed demonstration of the model. It is also essential to highlight the goal of the explainability aspect of the model. The visualizations showed to what extent certain factors contributed to the model’s decision, but that did not mean changing those factors would necessarily lead to a different outcome for the patient. For example, while the model might assign high importance to a certain treatment in the prediction of negative outcomes, it does not mean that not performing this treatment would lead to a better outcome.

Finally, all participant groups showed a preference for a more visual model. This matches the general consensus in visualization science, which argues for presenting numerical data in simple visualizations, such as bar or pie charts [15]. Our results showed that this is even more relevant for patients who have experienced a stroke. However, the visualizations that we used relied on red and green bars to highlight the importance of features, which can be an issue for colorblind people [34]. While none of our participants reported issues related to colorblindness, it should be adapted to a more inclusive visualization.

### Strengths and Limitations

One of the main strengths of this study was the involvement of different stakeholder groups, including patients, informal caregivers, and HCPs, with different roles. Moreover, the double setup with separate focus groups and questionnaires allowed for a check of agreement between the results of the different methods. One limitation to consider is our use of convenience sampling, which could have skewed the results. People with very severe stroke or with trouble reading or understanding were not represented. For HCPs, we mainly included people who were already open to research and new developments. The interviews with patients and informal caregivers were performed several months after the CVA, so their actual responses at the time of discharge might have been different. Moreover, we used mock-ups for the visualizations and not “real” predictions based on actual patient data. Although the visualizations were based on our existing models and data as much as possible, this was not a personal prediction. This made the visualizations more difficult to understand than expected, and the focus groups might have been more efficient with a functional model based on the actual patient data.

For the questionnaires, we had a relatively small sample size, and therefore, we did not perform any statistical analyses. As there was high agreement among the HCPs and the majority (9/16) of invited HCPs responded, it is likely they are a representative sample of the HCP group in the hospital, but we cannot know this for sure. Moreover, this group did not include all HCPs involved in stroke care. It is possible that physical therapists or general practitioners would have a different outlook on the prediction models, as they might be more involved in the rehabilitation of patients or involved at a different point in rehabilitation care. Lastly, this study was limited to a single hospital in the Netherlands. Differences between hospitals and especially between medical practices among countries can impact the generalizability of our results. Combining these limitations, we would recommend a quantitative follow-up study based on our questionnaire. Preferably, this should be a multicenter and international study to confirm the generalizability of our results outside the confines of our hospital.

### Implications and Future Directions

From this study, we can extract lessons to be considered when implementing AI in medicine in general. An important aspect is that the need for information provision is highly variable between groups as well as individuals. The results of the prediction model can be highly personal, and similarly, the needs of a patient and the setting and specific illness of the patient can be personal. It is essential to clearly define the purpose of the tool, as there are not only different levels of understanding but also different priorities. For example, patients do not always appreciate detailed information, especially if the information is not actionable but only informative. It is also essential to consider a model that can have multiple purposes. A model, such as ours, could also be used for advanced resource planning in rehabilitation clinics by estimating the future demand on the clinics and evaluating the effectiveness of the rehabilitation.

An important point is to investigate how to include patients who struggle with technology due to language, socioeconomic, or medical issues. For example, this study excluded non-Dutch speakers, but HCPs mentioned that language issues could further complicate explaining the expectations of recovery. This difficulty in communication should not be increased by a new technological tool or prediction model. Relying more on technology can lead to more inequality in care [35], especially for vulnerable patients, such as stroke patients [32]. One effort that can prevent such issues is the development of a comprehensive framework for AI implementation in health care and the creation of a roadmap for AI implementation. Such frameworks are being developed [31,33], but none of them are exhaustive, and having resources that detail considerations for AI application and evaluation are still necessary to develop AI with meaningful medical applications [33].

### Conclusions

The findings of this study show that participants are generally positive toward a prediction model for long-term outcomes of rehabilitation after stroke in CVA care under certain conditions, with a general preference for a more visualized prediction model. The prediction model should be geared toward HCPs, as they can provide the context necessary for patients and their informal caregivers. For HCPs, good reliability and relevance of the prediction model are essential for its proper integration. We recommend a quantitative follow-up study to confirm these results in multicenter and international settings.

## Appendix

Multimedia Appendix 1 Guide for focus groups with patients (translated from Dutch).

Multimedia Appendix 2 Guide for focus groups with health care professionals (translated from Dutch).

Multimedia Appendix 3 Finalized visualizations (translated from Dutch).

## Abbreviations

AI: artificial intelligence
CVA: cerebrovascular accident
HCP: health care professional
MIDI: Measurement Instrument for Determinants of Innovations
mRS: modified Rankin scale
