# Supplementary material for: The Significance of a Cerebrovascular Accident Outcome Prediction Model for Patients, Family Members, and Health Care Professionals: Qualitative Evaluation Study
Source: JMIR Hum Factors. 2025 Jan 22;12:e56521. doi: 10.2196/56521 (PMC11799809; doi:10.2196/56521)
Supplement: Multimedia Appendix 1 [file humanfactors_v12i1e56521_app1.docx]

## **Multimedia Appendix 1.** Guide for focus groups with patients (translated from Dutch).

Phase 1 *(What is the perspective of patients on the components and topics covered in the prediction model and what do they think of its use in the discharge interview?)*

- Explanation of research: The aim of this research is to see how we can improve the information provided to patients and their families about the expected course of stroke rehabilitation and recovery
- Introduction round:
  - Name, background, age, place of residence etc.
  - How did the rehabilitation period go for you?
  - What kind of rehabilitation, complications, how long was recovery?
- How did you experience the moment when the doctor provided information about rehabilitation during the discharge conversation
  - What did this moment of information provision look like?
  - Who gave you this information?
  - Did you understand the information you were given?
  - To what extent was there room for questions during this moment?
- Were figures (or data) used during the discharge interview?
  - For example: 75% chance of full recovery
  - If so, did you understand what these numbers meant?
  - If so, what kind of figures/data were these?
  - If so, how were these figures/data designed
  - If so, was it useful to have these figures/data at the discharge interview?
  - If so, did these figures/data make it clear
- Did you feel that your opinions/needs were taken into account when deciding on a particular rehabilitation plan?
  - Did you have a strong opinion and were asked for it, and at what point?
  - Who made the final decision?
  - Was there room in the conversation to provide input into the decision?
- To what extent was a personal long-term prediction made?
  - What was this prediction based on?
  - Was this prediction correct?
- Was it clear what you could expect from the rehabilitation in your personal situation?
  - If not, would you have liked this?
  - Did the rehabilitation period match the expectations you had or had been outlined?
- Have you used the CVA decision aid?
  - Why or not?
- Did you decide on a rehabilitation plan together with the doctor during discharge?
- What was most important to you in the discharge conversation?
  - For example: Amount of time in the rehabilitation clinic or rehabilitation at home, a suitable rehabilitation place, quality of life, long-term functionality

Phase 2 (Which visualizations of the stroke prediction model do stroke patients prefer, so that the goal is clear and understandable. Researchers shows and explain two types of prototypes to patients).

- Which prototype is the most insightful or understandable and why?
- Would such a prediction have changed your expectations?
  - If so, would you have needed such a prediction?
- Could such a prediction have influenced your decision about rehabilitation?
- Would such a prediction have helped with clarity during the family conversation?
  - And choice of rehabilitation institution
- Would such a prediction make the decision-making easier for you?
- Would you like to have a personal prediction for CVA decision aid, or would you rather discuss it with the healthcare provider?
