# Supplementary material for: The Significance of a Cerebrovascular Accident Outcome Prediction Model for Patients, Family Members, and Health Care Professionals: Qualitative Evaluation Study
Source: JMIR Hum Factors. 2025 Jan 22;12:e56521. doi: 10.2196/56521 (PMC11799809; doi:10.2196/56521)
Supplement: Multimedia Appendix 2 [file humanfactors_v12i1e56521_app2.docx]

## **Multimedia Appendix 2.** Guide for focus groups with health care professionals (translated from Dutch).

Phase 1

- Brief explanation about the research
  - Introducing ourselves
  - Explanation of the research & goals
- Short introduction:
  - Function
  - Work experience
  - Experience with discharge interviews and rehabilitation
- Do you have a good overview of expectations of the rehabilitation process of patients after discharge?
- What is the general provision of information about rehabilitation and long-term outcomes to the patient?
- Do you use data-based information (during the discharge interview)
  - Does this zoom in on the subgroup of the patient?
- Do you have enough information for the patient and informal caregiver about their individual situation and expectations?
  - How do you convey this information?
- Which outcome measure is most important to predict?
  - What do you and the patients value most?
  - Time in rehabilitation clinic
  - Quality of life
  - Functionality in the (medium) long term (3 months)

Phase 2

- Short explanation of prototypes
- Which of the prototypes is the most insightful and why?
- Which variables are important to show?
  - Premorbid score
  - Type of rehabilitation
  - Age
  - Sex
  - Complications
  - NIHSS on admission
- Would such a prediction help create more realistic expectations for a patient?
- Could such a prediction help patients and informal caregivers gain insight during the family meeting or discharge interview?
- When would this tool be used?
  - during discharge conversation
  - Link to CVA decision aid or in EHR
  - Later as additional information
