# Supplementary figures and images for: The Significance of a Cerebrovascular Accident Outcome Prediction Model for Patients, Family Members, and Health Care Professionals: Qualitative Evaluation Study
Source: JMIR Hum Factors. 2025 Jan 22;12:e56521. doi: 10.2196/56521 (PMC11799809; doi:10.2196/56521)

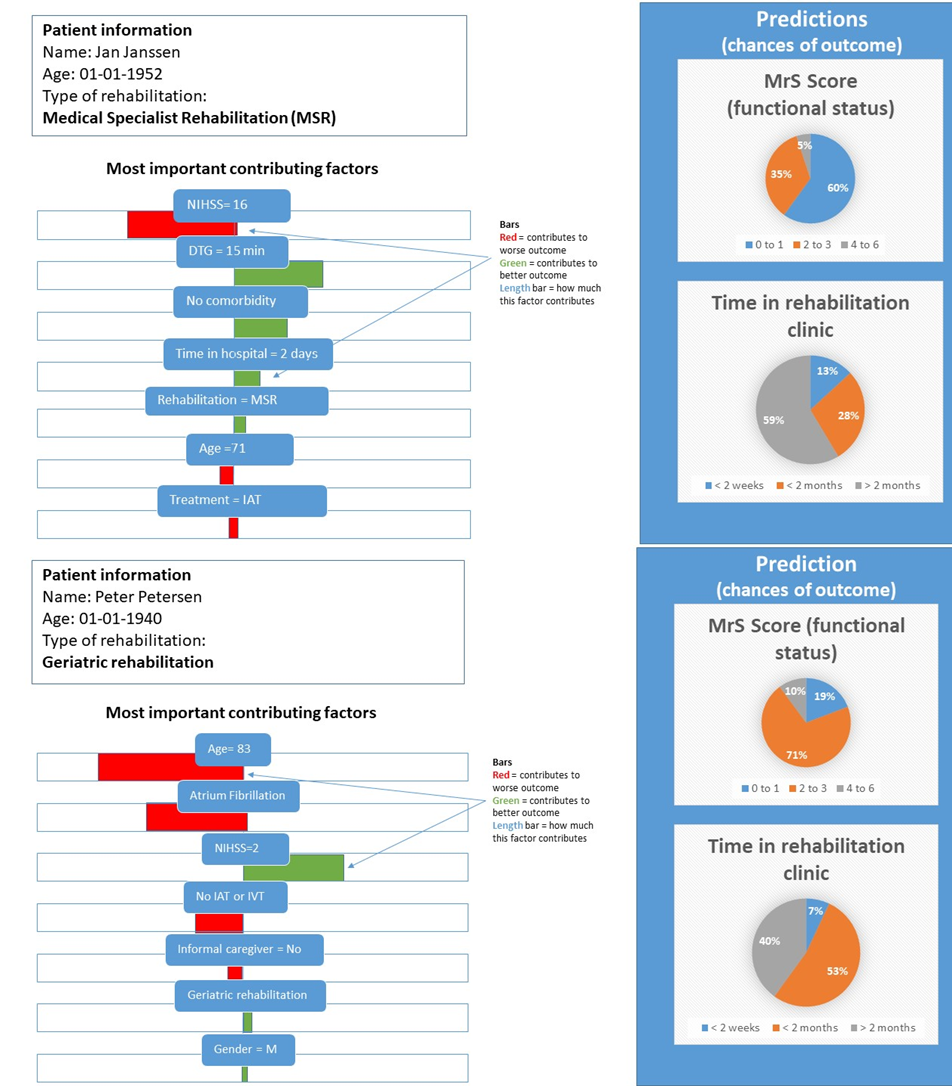

Supplement: Multimedia Appendix 3 [file humanfactors_v12i1e56521_app3.png]
